# Supporting Families to ‘Make the Most’ of the Relationship Between Their Companion Dogs and Their Children with Autism Spectrum Condition: A Qualitative Exploration of the ‘Family Dog Service’

**DOI:** 10.3390/bs15020162

**Published:** 2025-02-01

**Authors:** Emily Shoesmith, Heidi Stevens, Selina Gibsone, Cari Miles, Hannah Beal, Kelly Jennings, Elena Ratschen

**Affiliations:** 1 Department of Health Sciences, University of York, York Y010 5DD, UK; heidi.stevens@york.ac.uk (H.S.); elena.ratschen@york.ac.uk (E.R.); 2 Dogs for Good, The Frances Hay Centre, Blacklocks Hill, Banbury OX17 2BS, UK; selina.gibsone@dogsforgood.org (S.G.); cari.miles@dogsforgood.org (C.M.); hannah.beal@dogsforgood.org (H.B.); kelly.jennings@dogsforgood.org (K.J.)

**Keywords:** companion dogs, autism spectrum condition, mental health, qualitative

## Abstract

Evidence suggests that assistance and therapy dogs can positively impact the mental and physical health of children with autism spectrum condition (ASC) and their families. However, these services are often costly and inaccessible. As an alternative, companion dogs (e.g., non-specialist-trained pet dogs) may offer similar benefits. To support families in selecting and training a dog to benefit the whole family, a charity named Dogs for Good has designed the Family Dog Service. This qualitative study aimed to explore the perceived impact of human–dog interactions and relationships for children with ASC and their families, and their perceptions of the Family Dog Service. Interviews were conducted with 16 parents of children with ASC who had engaged with the service within the last five years. Thematic analysis identified four main themes. These related to the positive impact of the companion dog on the child with ASC (e.g., enhanced focus and attention, reduction in loneliness, and reduced anxiety-based behaviours) and the parents (e.g., increased respite and wellbeing). However, parents also reported challenges of dog ownership (e.g., adjustment to routines and barriers associated with the development of the human–dog relationship). These challenges appeared to be mitigated by tailored guidance and support from the Family Dog Service. The findings indicate that companion dog ownership provided benefits to children with ASC and their families, similar to those reported for autism assistance dogs. While the findings do not suggest that companion dogs can replace the important role played by specialist trained assistance dogs, it is possible that reported benefits may occur due to the development of the human–dog relationship, facilitated by the support offered by the Family Dog Service. The service may provide a practical, valuable alternative in light of restrictions and challenges often associated with securing assistance dog placements.

## 1. Introduction

Autism spectrum condition (ASC) is a heterogeneous neurodevelopmental condition defined by the DSM-5 as an individual experiencing persistent difficulties in social communication and social interaction across multiple contexts and exhibiting restricted and repetitive patterns of behaviours, interests, or activities (American Psychiatric Association, 2013). According to the Global Burden of Diseases, ASC impacted approximately 28 million individuals (Solmi et al., 2022) and nearly 3 million children under 5 years of age (Kang et al., 2023) internationally in 2019. As ASC is a lifelong and individualised condition that can impact education, employment, independent living, and social integration (Howlin et al., 2004), it is important that support is dynamic and adaptable to individual needs (Hall et al., 2016). Additionally, it is imperative that psychological and practical support is accessible to the primary caregivers of children with ASC (Hyman et al., 2020).

Dogs have been increasingly involved in support offered to children with ASC and their families in a range of contexts, such as dog-assisted services (Abadi et al., 2022; Galvany-López et al., 2024; Hill et al., 2020; Hüsgen et al., 2022; Nieforth et al., 2021) and assistance dog placements (Hellings et al., 2022; Leung et al., 2022; Shoesmith et al., 2024; Starkweather et al., 2024; Tseng, 2023). Evidence indicates that dog-assisted services can have positive impacts on both the mental and physical health of children with ASC, including improved social behaviour and interaction (Ben-Itzchak & Zachor, 2021; Nieforth et al., 2021), behavioural regulation (London et al., 2020), physical activity (Abadi et al., 2022), and reduced stress (Meints et al., 2022). Likewise, previous research suggests that assistance dogs are able to support children with ASC with their emotional regulation, community participation, and social skills (Appleby et al., 2022; Burgoyne et al., 2014; Carlisle, 2015; Hellings et al., 2022; Shoesmith et al., 2024; Tseng, 2023), as well as enhance wider family wellbeing (Hellings et al., 2022; Leung et al., 2022; Shoesmith et al., 2024). However, further research is needed, as earlier studies on dog-assisted services or assistance dogs for children with ASC are limited due to variability in methodologies, implementation, and reporting (Tseng, 2023).

While it is increasingly well established that assistance dogs and certified therapy dogs can be beneficial for children with ASC and their families (Abadi et al., 2022; Ben-Itzchak & Zachor, 2021; Hellings et al., 2022; London et al., 2020; Shoesmith et al., 2024), this support is often inaccessible and expensive (Hall et al., 2016). Therefore, companion dogs (e.g., non-specialist-trained pet dogs) might be a potential, more practical alternative. Indeed, research has reported that similar benefits for children with ASC might be accrued from companion dogs without specialist training (Wright et al., 2016); for example, improved prosocial behaviours (Grandgeorge et al., 2012), reduced restrictive behaviour patterns (Byström & Persson, 2015), and reduced anxiety (Wright et al., 2015b). Additionally, companion dogs can reduce stress for primary caregivers (Wright et al., 2015a) and enhance overall family functioning (Walsh, 2009; Wright et al., 2015b). However, there are also challenges associated with dog ownership, including the time commitment for training and care, potential issues with the child bonding with the dog, risks of dog bites, and financial costs (Carlisle, 2014; Wright et al., 2016). Therefore, it is imperative for families to consider both the benefits and challenges of dog ownership, and how to establish a healthy relationship between the dog and family members.

To equip parents with the knowledge and long-term support required for choosing and training a dog, a UK-based charity named Dogs for Good has designed the Family Dog Service. The workshops involved in this service aim to harness the full potential of a companion dog to improve the lives of children with ASC and their families, while prioritising the welfare and wellbeing of the humans and dogs involved. While research has explored the impact of assistance and companion dogs on various populations, including children with ASC (Abadi et al., 2022; Ben-Itzchak & Zachor, 2021; Hellings et al., 2022; London et al., 2020; Shoesmith et al., 2024), there have been no investigations into the effects of any novel support and educational programmes designed to help families select and train a companion dog for families with ASC. Therefore, the aim of the current study was to explore the parental experiences of the Family Dog Service and the perceived impact of human–animal interactions and relationships on children with ASC and their families. This study offers entirely new insights by examining how such a service supports the parents of children with ASC across different stages of dog ownership.

## 2. Materials and Methods

### 2.1. Study Design

Individual qualitative semi-structured interviews were conducted with the parents of children with ASC who had engaged with the Family Dog Service.

### 2.2. Ethical Approval

Ethical approval for the study was granted by the Health Sciences Research Ethics Committee at the University of York, UK, on 21 November 2023 (ref: HSRGC/2023/596/B).

### 2.3. Overview of the Dogs for Good Service

The service is a UK-based charity named Dogs for Good (previously known as Dogs for the Disabled). This charity provides a range of services including the training and placement of assistance dogs with adults and children (Shoesmith et al., 2024). Dogs for Good also offer the Family Dog Service, open to parents of autistic children (aged between 3 and 16 years), including those currently on the diagnostic pathway). The broad age range ensures that the service is accessible to a wide demographic of families with children with ASC, thereby maximising potential reach. The workshops are designed for families at every stage of dog ownership, including the following: (1) the early stages, when families are considering and researching how a dog can support their child; (2) the process of choosing, introducing, and training a dog; and (3) those who already own a companion dog and are seeking guidance on how the dog can support both their child and the wider family.

The Family Dog Service began in June 2010, and their workshops were delivered in-person until 2020. In September 2020, the service transitioned to online workshops due to the COVID-19 pandemic. To deliver this service remotely, the Family Dog Service’s workshops are pre-recorded by dedicated Family Dog Instructors and accessed via the online platform SharePoint. Each pre-recorded workshop is accompanied by a corresponding ‘workshop live’ event, held as a group video call on Microsoft Teams. The ‘workshop live’ events are led by dedicated Family Dog Instructors and recap the pre-recorded workshop content, offer the opportunity for the instructors to answer any questions, and generate discussions within the group. Five ‘workshop lives’ are available with a duration of 1.5–2 h, with the exception of the introduction that is approximately 45 min (Table 1).

In addition to the workshops, families also receive access to lifetime support from the Family Dog Instructors and a range of resources including fact sheets and videos. These resources provide supplementary guidance and information to what is covered in the workshops. Additionally, families have access to a private Family Dog Service Facebook group to seek advice or support from the instructors and their peers, and this group is moderated by the dedicated Family Dog Instructors.

### 2.4. Recruitment

An advert to invite expressions of interest in participating in this study’s interviews was posted in the private Family Dog Service Facebook group. The private Facebook group is only accessible to members who have completed (or are currently completing) the Family Dog Service workshops. Contact details were provided on the advert, so interested members could contact the researcher directly. Prior to obtaining their consent, the first author responded to potential participants to confirm they met the inclusion criteria: (1) have at least one child with ASC aged 3–16 years (including those on the diagnostic pathway); (2) be current or recent (last 5 years) participants in the Family Dog Service; and (3) be able to speak English sufficiently to participate in interviews. Eligible participants were sent a Participant Information Sheet and consent form to complete and return via email.

### 2.5. Data Collection

Demographic information was gathered about participants’ age (in bands), gender (male, female, non-binary/third gender), number of children with ASC (confirmed or suspected diagnosis), their age (in bands), gender (male, female, non-binary/third gender), and any comorbidities (confirmed or suspected).

Semi-structured interviews were conducted by the first author to gain an in-depth understanding of participating families’ experiences with the Family Dog Service, and the perceived impact of human–animal interactions and relationships for children with ASC and their wider family network. Interview topic guides were developed based on existing posts in the Family Dog Service Facebook group. Data were obtained from posts in the Family Dog Service Facebook group and imported to Microsoft Excel. A thematic coding framework was generated that consisted of categories synthesising the experiences of the service and/or the family dog that participants described. This framework facilitated the development of the interview topic guide, which was then refined in collaboration with the Dogs for Good team.

The interview topic guide is provided in Supplementary Material S1. All interviews were conducted via a remote platform, were audio recorded and transcribed verbatim. All participants were given ID numbers to anonymise the transcripts. Data collection took place between January and April 2024.

### 2.6. Data Analysis

Thematic analysis was conducted, including data familiarisation, coding, and searching for and defining themes (Braun & Clarke, 2006). Data collection and analysis occurred concurrently; themes and issues from earlier interviews informed the content of subsequent interviews, and the topic guide continued to evolve throughout data collection. As data collection and analysis progressed, a coding framework was devised and applied to transcripts by ES using NVivo 13 (Lumivero, 2020). A second author (HS) independently reviewed the themes to ensure consensus with the assignment of theme names and illustrative quotes.

## 3. Results

Interviews were conducted with 16 parents who collectively reported about 25 children with ASC (Table 2). The average length was 51 min (range: 35–68 min).

The thematic analysis of transcripts resulted in the identification of four main themes with associated clusters of sub-themes related to various aspects of human–animal interactions and relationships, family dynamics, and experiences of the Family Dog Service (Table 3). To illustrate themes and sub-themes, verbatim quotes are presented below.

### 3.1. Improvement of Psychosocial and Functional Aspects Associated with ASC

#### 3.1.1. Enhanced Focus and Attention

Many participants reported that their child’s focus and attention had improved since integrating the dog into the family unit. Participants expressed how their child was more willing to engage with and maintain concentration on activities due to the presence of the dog.

*“His attention is much more focused with her, being able to act the responsible owner. Sometimes, he is hyper focused on his computer, and he doesn’t need [dog’s name] around him, and that’s fine, but when he’s not, she certainly increases his focus on activities that she’s involved with, and that’s where she pays dividends again. She’s fantastic.”*

*“Since [dog’s name], his academic ability has come on. We try to get [dog’s name] to listen to him read, I think it is knowing somebody is there on his side as well. So, it’s really helped his language because he likes to read to [dog’s name], and it keeps him focused.”*

The responsibility associated with dog ownership also appeared to improve the child’s focus and willingness to partake in caretaking tasks (e.g., walking, grooming, and training the dog).

*“He loves the fact he’s got something to focus on, someone to look after, because most of the time, it’s other people keeping him on track, so this is the other way around, and I think he likes that.”*

*“The focus is on, you know, I want to give her food, I want to see her, walk her, and give her cuddles before school, I want to get back from school because I want to see what she has been up to.”*

Additionally, one participant spoke about how their child frequently did not respond to a range of stimuli, including animals in community settings. While this caused initial apprehension to introduce a dog to the home, the parent noted how their child had shown improvements and started to pay attention to the dog’s activities.

*“Whenever we go to zoos or farms or anything, he does not notice any animals and that’s why we were worried about getting a dog. But he notices [dog’s name]. He watches him, he sees what he’s doing, and sometimes, if he’s gone into his crate, [son’s name] will lean over the sofa and look around to see where he’s gone. He’ll like, lean up to see where he is, which is lovely.”*

#### 3.1.2. Reduction in Loneliness and Engagement in Social Interaction

Parents reported that the constant source of companionship appeared to ameliorate the child’s feelings of loneliness. This was often attributed to the positive relationship established between the child and the dog.

*“[Daughter’s name] feels lonely, and I think she’s isolated because she’s not accessing school […] I can already see there’s a friendship there with [dog’s name], like an unconditional friendship, and I think when [daughter’s name] is feeling lonely, she can go to see [dog’s name] and it’ll take that feeling of loneliness away. I think the loneliness aspect, it’s not quite the same as having, like, a girl friend, but I think it still makes her feel like she’s got somebody, that social companionship. I think a dog can bring out so much and make a world of difference.”*

*“They’re just best friends. As soon as [daughter’s name] wakes up in the morning, [dog’s name] is straight up there to say hello, just having that somebody who’s happy to see you with a big waggy tail. It’s about the companionship, it’s her best friend. They do everything together. Just having that for [daughter’s name] is amazing. It helps bring her down, it’s somebody that she doesn’t have to explain that she’s in a mood to, [dog’s name] is just there with a happy little face, I think it’s that utter acceptance that a dog brings. The bond between them is really strong.”*

Many participants also noted that they had observed an improvement in their child’s social interaction. Specifically, they expressed that their child’s confidence had improved when interacting with others when the topic of conversation focused on the dog.

*“[Daughter’s name] is selective mute, she doesn’t speak very often unless she’s really calm at home. The dog helps her to talk because she’ll print out pictures of the dog, she made an album and printed it all out and took it to show all the teachers, and it opens the conversation, and then she’d able to answer questions about the dog, which is really amazing.”*

*“I think [daughter’s name] doesn’t mind if people come over and talk to her about [dog’s name] because it’s not about her. She’s found that deflection quite good, because it’s something she knows about. She’s my dog, so I can talk to you about it.”*

Lastly, the presence of the dog appeared to represent a shared common interest, facilitating discussions and aiding conflict management in a way that did not cause distress or overwhelm the child.

*“The dog is an icebreaker for us, because if I tell [son’s name] to do anything, he won’t like it. He can become aggressive towards commands, but with the dog, he likes to speak about it, he will be much better, he will talk to us about it, if we are talking about the dog while asking him to do something, that’s better. It’s like a buffer between us.”*

*“It gives us a common conversation, a different conversation, whereas for the last 12 years, it’s been about her behaviour, it’s been about the meltdowns. She doesn’t want the attention in that way but that’s what the conversation has been about. The fact I can train [daughter’s name] to train [dog’s name] through what I’ve learned through Dogs for Good, I think that’s great.”*

#### 3.1.3. Increased Physical Exercise and Time Outdoors

The majority of participants spoke about how the family dog had improved the child’s motivation and willingness to exercise and spend time outdoors. While this was directly beneficial for the child’s fitness and activity levels, most participants reported how this had been valuable for their child’s wellbeing and emotional regulation.

*“We play rugby after school, and he goes through phases where he doesn’t want to go at all, and when he didn’t want to go, I suggested [dog’s name] comes with us, and then he was happy to go. So, it actually helped us to get out and do that because physical activity is also really good for him to regulate.”*

*“She would never, even though she probably needed to get her tunes in and walk around the block after school to get rid of the day, she would never say, can I go for a walk? Now, she’ll come in, play with [dog’s name], and say, I’m taking him for a walk, and off they go. You just don’t realise how revolutionary that is until you stop and think, she’s just left the house on her own! Just having that for her mental health, getting outside, having the exercise.”*

Additionally, one participant spoke about his child’s hypersensitivity to light, which affected their confidence and motivation to spend time outdoors. However, this had improved *‘an incredible amount’* since owning the family dog.

*“We’ve had some really great successes with him being able to go outside […] he struggles with changes of lighting, so going outside is such a big thing for him, and he will really struggle. He’s got ski goggles he wears and photochromatic glasses, and he’s got a way of going out effectively, which he can do, but it’s helped an incredible amount having [dog’s name].”*

#### 3.1.4. Reduced Anxiety-Based Behaviours

All participants spoke about how the family dog provided a calming influence on their child with ASC, particularly when they were distressed and exhibited anxiety-based behaviours.

*“It is just so clear he feels much safer and comfortable with just her presence. If he has a wobble, he just focuses on [dog’s name], even just a pat on the head, it really reduces his anxiety that was much more heightened without her.”*

Parents reported that children with pathological demand avoidance (PDA) often responded negatively to demands or expectations, resulting in heightened anxiety. Participants expressed that their dog’s non-judgemental and predictable behaviour helped to soothe the child, particularly during stressful episodes.

*“With his PDA, he struggles with anything that feels like a demand. Even something as simple as getting dressed can trigger a meltdown. But with [dog’s name], there’s no pressure. She’s just there, waiting calmly, and somehow that helps him regulate himself. When he’s stressed, she doesn’t demand anything from him, she just offers comfort. It’s like she knows exactly what he needs without asking for anything in return.”*

Some participants reported that their child had difficulty with behavioural and emotional regulation that could lead to violence, withdrawal, or avoidance of social and sensory experiences. The calming effect and unconditional love from the dog were noticeable when daily stress was experienced.

*“I think it’s that unconditional love. I think for [son’s name], with his PDA, he has a lot of shame and guilt when he’s had meltdowns, when he’s hurt people. He says, ‘I’m really sorry, I’m really awful, that was awful of me, I hate myself’. It’s very unconditional for [dog’s name], he doesn’t hold a grudge even if he does have a massive meltdown right next to him, [dog’s name] doesn’t even react to [son’s name], he will just snuggle into him for a cuddle, or for him to give him a stroke. So, he’s a constant in that respect, there’s no judgement, there’s no kind of, I just want to be with you when you’re happy, I just want to be with you when you’re quiet.”*

Some participants expressed how their dog would seek closer proximity to their child during a meltdown and would often *‘intuitively’* know what the child needed to comfort them. This could include laying on the child, other physical contact, or simply being present in the same room.

*“She’s fantastic when [son’s name] is having a meltdown, she knows. Seems to just know whether he needs her near him, not near him, touching him, not touching him. Once, he started hyperventilating […], his legs were thrashing all over the place. Without being instructed to do anything, [dog’s name] very slowly sort of pulled herself and laid over his lower leg and pinned his legs down. She was very calm about the whole thing, just very slowly, put a foot and then a head over his ankles, and within about five minutes, his whole body relaxed.”*

*“She goes to [daughter’s name], even if she’s crying and she’s really loud, [dog’s name] goes to her and wants to help […] leaning against her, or something like that, you know, just a presence, a calming presence.”*

*“[Daughter’s name] has meltdowns, but actually, we’re finding with [dog’s name] here, it almost prevents them, because she’s there ready for a cuddle, and that just helps calm everything down. She’s very tactile, so the minute you sit down, you get this 15kg dog literally jump on you, and that at the moment is enough for [daughter’s name].”*

Additionally, participants spoke about how their child benefitted from the opportunity to verbalise their anxieties to the dog without judgement.

*“[Daughter’s name] might worry about telling me something, yet I can hear her chatting to [dog’s name] upstairs, and I think [dog’s name] has changed the dynamic, and has been something to diffuse whatever is going on for her, I didn’t think I would see the benefit, because how could possibly a dog take that all away? But she does. [Daughter’s name] still speaks to me, obviously, but it’s not that constant, ‘mum, I need to be with you, because that’s where I feel safe’, that’s kind of deflect to [dog’s name], where she feels her version of safe, she says she feels emotionally safe.”*

*“If he’s had a big meltdown, he finds it really difficult to then recover from that and not feel really shameful and really guilty. I’ve noticed he will go upstairs with [dog’s name] and I hear him talking to [dog’s name] and telling him, ‘I need a cuddle, I’ve been such a naughty boy, I’m such a horrible person. I guess if he didn’t have [dog’s name], he wouldn’t have anyone to do that with because he doesn’t want to do it with us because he’s still feeling very bad about it. It’s nice for him to be able to externalise that more than keeping it all in.”*

Lastly, while some participants stated that they wanted to train their dog to specifically prevent a meltdown or provide deep pressure therapy, the companionship provided by the dog, the development of the human–dog relationship, and subsequent enhanced wellbeing was frequently seen as the priority.

*“You finally feel like you’ve got some help for your child, but you have to remember it’s a dog. I put so much hope into [dog’s name] to help me with [daughter’s name], but I still need [dog’s name] to be happy too, and first and foremost, she’s a family pet. She’s a family pet, and like I say, if eventually she doesn’t do deep pressure therapy, but she still gives her great cuddles and flops all over her, fine, you know, she’s not going to be kicked out or not pass an exam. You know, she’s good enough already, so, great.”*

*“I didn’t anticipate the impact she has truly had. I just thought I was going to be able to train her for pressure therapy ultimately, but I didn’t anticipate the overarching support mechanism that she was going to become for both [son’s name], and if I’m really honest, hand on heart, myself. It’s that sort of bond, and something else to focus on.”*

#### 3.1.5. Tolerating Changes and Willingness to Engage in New Activities

Some participants noted that the family dog had influenced their child’s acceptance to change and willingness to try new things. For many parents, this was an important achievement given their child’s usual fixed interests and rigid behaviours.

*“When you go on holiday, you’re not really sure what you’re going to do for the day, my kids can’t deal with that because they don’t know where they’re going. They don’t know what to expect, we haven’t planned it. It’s really hard for them. Put the dog into the mix, and they ground us, everyone’s so much calmer, and we can just chill.”*

*“[Son’s name] would never go downstairs by himself. Now he knows [dog’s name] is there, he’s really happy to go downstairs in the morning, he’ll go get [dog’s name] and watch some TV, whereas before, he wouldn’t have done that, he’s happy to do that now because he knows [dog’s name] is there, it’s amazing.”*

One participant suggested that their child’s acceptance to change could be attributed to the sense of responsibility associated with ownership, and the child understood the requirement to fulfil the dog’s needs.

*“When [dog’s name] needs a walk, that sort of, overrules his needs and overrules the ‘Mummy needs to be home at this time’, you say, well, who’s going to walk [dog’s name]? And then if he knows she needs a walk, that’s okay, he understands that.”*

### 3.2. Positive Impact of the Companion Dog on Parents of Children with ASC

#### 3.2.1. Increased Parental Respite

In addition to the direct benefits of the family dog to the child with ASC, the majority of parents expressed that the dog also had a positive impact on them. Specifically, participants indicated that they felt less stressed, as their child felt safe and comfortable in the presence of the dog, and this subsequently led to an increase in respite for the parent.

*“She’s taken the pressure off. [Dog’s name] is an extension of me, but in a way that I could never give [daughter’s name]. I didn’t realise how much I needed her, not for cuddles or whatever, but I needed her to help me to help [daughter’s name], to take some of that pressure off, to take some of that emotional burden away, which is a big ask for a little yellow Labrador, but actually [dog’s name] does that, just by being there. So, that then directly impacts the whole house, because everything is just that little bit calmer. I have been [daughter’s name] emotional crutch, and it’s now given me some time to myself.”*

Some participants reported that before they introduced a dog to the home, they experienced feelings of guilt when taking time for themselves. However, dog ownership allowed them to take *‘justifiable respite’* while meeting the needs of the dog, which appeared to reduce these negative feelings. Participants indicated this benefit was often unexpected as they had introduced the dog to the home primarily to support their child with ASC.

*“The thing that I didn’t expect was actually me having some time outdoors by myself. Having some time to myself in the fresh air, and for it to be justified, because when I used to work, that used to be my respite. But it was justifiable respite, because it was bringing money in and helping other people. This has given me that again, because I can justify it, I’m meeting his needs as well, but then I’m also getting time to myself.”*

One participant also spoke about the benefit of the dog when their child was with a professional caregiver, as she was able to spend time outdoors with her partner and the dog, which increased feelings of normalcy.

*“When we’ve got a carer over for [son’s name], we’ll take [dog’s name] to the pub, he’ll sit with us in the pub. It’s just a nice thing, like, we can actually be normal and do normal things because we can’t ever take [son’s name] anywhere like that. It’s nice to go out as sort of a family with our dog child and sit and have a drink, and just feel normal. I think that’s a massive thing because that just doesn’t happen in day to day life.”*

#### 3.2.2. Increased Parental Wellbeing

Many participants also spoke about how the dog had increased their own and their partner’s mental health and wellbeing, primarily attributed to the unconditional love and companionship provided by the dog. This also appeared to be an unexpected benefit, as participants indicated that this improvement in wellbeing was surprising.

*“For us, it was unexpected. I didn’t ever think about it from my point of view other than the additional burden. I know that sounds horrible, but I was doing it for the kids […] I did not at any point think about the companionship and bond for me or [husband’s name]. I just didn’t even think about that, and how good it makes us feel, so that has been a pleasant surprise.”*

*“I never considered the positive impact of just having her. It’s been a pleasant surprise, and we find it so rewarding. Just to have a cuddle, that unconditional love, like this morning, when I woke up, I felt sad and heavy, and [husband’s name] came up with [dog’s name], which normally we don’t do. She just got on the bed with me, and it was like, okay, I’m okay now, I’m going to get on with the day. So, she is benefitting us.”*

### 3.3. Considerations and Challenges Associated with Dog Ownership for Families with Children with ASC

#### 3.3.1. Key Considerations When Selecting a Dog

Participants identified important factors to consider when selecting the correct dog breed for their family, including size and sensory issues (e.g., coat length, type).

*“I needed a dog of a fair size because we’re doing deep pressure therapy with [daughter’s name], and I learned that, you know, the heavier the better. Probably the Labrador was as heavy as I was prepared to go.”*

*“My son wouldn’t have coped with dogs that either moult or shed a lot, but then golden retrievers were talked about as being really good. So, he doesn’t like wiry sort of coats. So, the golden doodle just ticked so many boxes. You know, that doesn’t shed and has got a long, shaggy coat.”*

Participants frequently noted that they may not have thought about these factors if they had not completed the Family Dog Service workshops.

*“It was really helpful, and I think it’d be really helpful for a lot of other people, especially because things like, what kind of preference does your child have to touching things, and like, are they sensory seekers? Are they going to be kind of rough handed with the dog? You know, do you need a bigger dog or like, would a smaller dog be better? I think there were so many questions that were really interesting things I never really would have thought about.”*

#### 3.3.2. Adjusting to a New Dog Within the Family Unit

Participants often spoke about the challenge associated with integrating a new dog into the family unit and adjusting to a new lifestyle and routine. This challenge was amplified in certain situations, for example, when the child was described as less tolerant to change or if the child had germ phobia.

*“Initially, he was terrible. He wouldn’t touch her. If he did touch her, he had to wash his hands. He would stand and watch me bath her but wouldn’t involve himself. He would put her food on the floor but wouldn’t prepare the food. Whilst we were house training, that was very challenging as he would laugh and giggle if she did her business inside, but it really did kick in as soon as it was tidied up, it was, has it been disinfected? What have you wiped it with? Is that clean? Can I walk on it?”*

*“There was obviously the changing routine, and it did impact the kids a bit, particularly [daughter’s name] who did struggle with something else coming ahead of her, like, if his needs needed to be met first before hers.”*

The change in routine also impacted some parents, who highlighted that the responsibility of caring for a dog was more challenging and time consuming than anticipated.

*“I found it more challenging than anyone because it was my routine that changed the most, it changed my life, completely. I’m the one who has to rush back from work, I’m the one who gets up to go out early in the morning, even if I’ve got something on. I knew it was going to be hard, and it would be a big commitment, but it’s probably bigger than I ever realised.”*

As a result, participants identified that this sometimes compromised the way families felt about acquiring a dog, but strategies and support provided by the Family Dog team appeared to mitigate this barrier. Subsequently, this appeared to enhance the relationship between the family and the dog and ensure that the integration and lifestyle adjustment was more manageable.

*“It’s quite tricky at the beginning, it’s hard work, and I’d be on walks, and I was like, nearly crying, because she’s pulling so much. I was like, oh my goodness, what have I done? It was helpful to speak about focusing on like, how it will be able to help [son’s name]. They also include stories in there to explain how it’s helped other families too, and it’s really good, because that helps you to sort of, get through the hard times.”*

*“It can be a really challenging start, and I think it was so useful because they like, help to set her up to succeed, and setting up this relationship to what will work. You know, like positive reinforcement, not putting too more pressure on the relationship between them, giving ideas for stuff like enrichment that [son’s name] could get involved in, you know, like, little puzzle toys. [The service] helped me to introduce them in a certain way and I wouldn’t have known how to do that.”*

#### 3.3.3. Challenges Associated with the Development of the Human–Dog Relationship

While many of the perceived benefits to the child with ASC were attributed to the development and impact of the human–dog relationship, the majority of participants described that this relationship took time to develop.

*“I’m not going to say there was an instant bond. Everything within him wanted to adore her like he could see people around him doing. But he does take a while, but it’s because he approaches everything from a logical perspective and not an emotional one, but that almost is the barrier that prevents him from being able to relax and enjoy things sometimes.”*

Participants highlighted barriers to the development of the relationship that included impatience from the child when training the puppy, behaviour associated with a puppy (e.g., nipping, jumping) that distressed the child and contributed to sensory overload, and germ phobia.

*“[Daughter’s name] was so fixated on her being there, and we had to say, puppies sleep, you’re not going to pick her up and put her on you when she’s sleeping. It was getting [daughter’s name] to settle, because the anticipation was so much and she just wanted this dog to help her, and we had to slow her down and keep in mind she is still only a puppy.”*

*“At the time, she just thought [dog’s name] doesn’t like me, and we had to explain that she’s done it to all of us. But obviously, [daughter’s name] reaction is much stronger to getting a little scratch from a puppy tooth and we can just go, it’s just a little scratch, but for [daughter’s name], little scratches are more painful or a bigger sensory experience.”*

*“In the first two weeks, [dog’s name] was sneezing phlegm balls out, and he hates having like anything gross on him. So, her sneezing all over him, luckily, it did stop after two weeks, but that added to it, like, he didn’t really want to be near her in case she sneezed on him, so that was hard too, but who can plan for that?”*

While the development of the relationship was often perceived as a time-consuming but worthwhile process, many participants expressed that the Family Dog Service provided support and advice that helped to build and maintain the relationship.

*“The bond is growing, it’s certainly progressing. The service has definitely helped, all the things like setting up at the beginning to help not put pressure on him and the relationship between them […] it’s definitely better because of the service, because it’s helped me to introduce them in a certain way and I wouldn’t have known how to do that.”*

*“I think [the relationship] would have naturally occurred, but I think what it’s done, it’s sort of sped it up or just gave us a bit more insight into what’s possible. I think if we just got a dog and did normal puppy training, we would have seen benefits, just from having a dog who has an affectionate and sociable nature. But, what the Family Dog Service did, is kind of, help us realise what is possible and sped that up […] I think it helps to, that sort of hand holding and being guided on the path of, this is what is possible with a family dog to support a child with autism.”*

### 3.4. Experiences of the Family Dog Service Workshops

#### 3.4.1. Motivation to Attend the Workshops

The majority of participants expressed that they were motivated to engage with the Family Dog Service as they had initially embarked on research relating to autism assistance dog placements. However, many parents identified barriers to these placements, including the expense, the strict criteria related to their child’s age and stage of diagnostic pathway, and the time taken to be matched with an appropriate assistance dog. Therefore, many participants spoke about how the Family Dog Service was an appropriate and beneficial alternative.

*“We wanted to get a dog from a charity, fully trained, arrive on your doorstep, here I am, but because of lockdown, we kind of missed the boat on that as [son’s name] was getting too old. I think he was 10 by the time we made a definite decision, and most of them had shut their books. So, that was kind of like, if we’re going to do it, we’re going to have to do it ourselves. How can we do that? So, I looked what was available to try and understand if it was really doable because [son’s name] is quite challenging and a lot of hard work, so I wanted to make sure I was going to be able to manage that alongside looking after him as well.”*

Other parents expressed that they signed up for the service to gain support and advice about introducing a dog into the family, especially for those who had no previous experience of dog ownership.

*“We didn’t have any experience with dogs, we didn’t really know what we were doing. So, just having somebody to kind of support us and help set us up right, so we can get the right outcomes that would help, that would work for us, because it was a bit risky in the sense that like, it could go terribly wrong. The dog could just be barking all the time, and it could have a really negative impact, so it was kind of having the knowledge to know what we were doing to set her up, and set her up right, to support [son’s name].”*

Additionally, all parents expressed that the workshops appealed to them as the workshop content was tailored to families with ASC, and that in itself was motivation to sign up.

*“What I’ve found is, with an autism diagnosis, it’s like a full stop. You get your report, you have all this hype and so many years of building up to it, and then that’s it. We had to self-educate very much. One part of that was through a lot of Googling, what support can I get for my child with autism? […] I went on their website and they’re very much family-oriented and work with families with autism. I almost wanted to cry with relief after finding them.”*

#### 3.4.2. Information Tailored to Families with ASC

All participants spoke about the value of the workshops offering information tailored to families with ASC. In particular, participants frequently expressed the benefit of discussing ways to avoid sensory overload, or the benefit of sensory feedback from dog-related activities.

*“They talked about, the sound of the dogs, I mean, this is so specific, but the sound of the dog’s collar, the dog’s tag clinking against the dog bowl can be a sensory issue, and for us, that is definitely true. I hadn’t thought of it, but it definitely is, and so they explained that you don’t have to feed your dog that way. There are lots of different ways you can do it. You know, you can scatter feed, you can use a lick mat, it just absolutely blew my mind.”*

*“It’s definitely helped us to think about things and to see there are other ways of doing things, like the sort of sensory specific stuff, they did a really useful section on that […] They talked about using a particular grooming tool so that your child gets sensory feedback from grooming, but also is good for the dog, you know, things I didn’t think about.”*

Participants provided other examples of how the content was tailored to families with ASC, including support and strategies to facilitate the introduction of a new dog into a family unit with children with ASC.

*“They talk about how to have a dog in the household when you’ve got a child who is autistic and how to help them to relate to the dog because they might not necessarily have that understanding. They said a few things that stuck in my mind, like having a collar or bedding that was related to the special interest of the child, so they felt like the dog was more connected to them.”*

*“They suggested to send [the breeder] audio clips of him having a meltdown so [the breeder] used to play them to the puppies when they were tiny, alongside washing machine noises and you know, the kind of normal socialisation bits that a good breeder might do. The first meltdown he had was the day after he arrived […], he just trotted over to the same room as [son’s name], sat down next to him, and just didn’t even bat an eyelid. He was not fazed at all, and I think [son’s name] was so shocked by that because [dog’s name] had opted to be there, opted to be with him.”*

In addition, the content to support parents to train the family dog ‘advanced task work’ was well received, as these skills would have a positive impact on the child with ASC.

*“We did all the conventional training courses with our springer spaniel, but this is more for that interaction with the family, and you know, with the children with autism. Particularly the nose nudge and the head rest, those will be the techniques that are really helpful for children with autism, but you wouldn’t get that on a conventional training course.”*

Many participants identified how the Family Dog Service may differ from a conventional dog training course. They expressed that the tailored content and support would likely not be provided in ‘traditional’ training, as the focus would be on training the dog rather than training the dog to support the child with ASC.

*“It’s the understanding of the unique nature of autistic families, and how a lot of other training courses would just require you to make changes to how you behave and interact with the dog, for example, telling your children that they can do this, and they can’t do that, that just doesn’t work for us. We need somebody who understands that our children don’t work that way, and they might need, you know, tailored resources. It’s the fact they combine up to date modern, sympathetic training methods with the understanding of the particular challenges that our families face, combining the two together, it’s just amazing.”*

Lastly, parents also appreciated how the service was tailored to families with ASC while still prioritising the welfare of the family dog, promoting the use of positive reinforcement, and treating the dog as an equal member of the family. This included considering the dog’s safety and wellbeing, especially when the child with ASC exhibited anxiety-related behaviours.

*“It hugely helped us to prepare, knowing how to manage a dog during a meltdown, it was so important to get right from the start, so we don’t scare [dog’s name]. Thinking about giving him places to go to rest if [son’s name] is wound up. They also taught us so much about socialising and dog behaviour which means I think we are getting more out of our dog than we would have.”*

*“It was all about setting the dog up to succeed, like everything we did in those early stages taught her so much with regards to self-control and it just made it all work, it was just all the building blocks for her to succeed. I think because it was all positive reinforcement as well, it was just really nice and refreshing, it just really worked for us.”*

#### 3.4.3. Access to Lifetime Support

All participants spoke about the value of the lifetime support offered by the Family Dog Service team. Many participants expressed how they had contacted the team between or upon completion of the workshops to seek additional advice or support and always received detailed responses which answered their queries and provided reassurance. Additionally, there was consensus among participants that these responses went above and beyond that of independent research due to the team’s knowledge and expertise about both dog training and supporting families with ASC.

*“The thing that was most attractive for all of it was how approachable the team were, and the fact they are always there if you’ve got a question. They were very clear, if at any point you need any support on training your dog, you’ve got any questions, no matter how daft, please get in touch with us, we will always help you. I think because autism is not something that is a static condition, knowing how hard it is to get any kind of support for an autistic child, it was beyond comforting to know that they were probably one of the few people I’ve ever spoken to where they’d actually said that, don’t worry if things change, and as and when they change, we can help, we can always work with you, look at behaviours and see how we can build something in.”*

In addition to the ongoing support offered by the team, participants also have access to ongoing peer support via the private Facebook group. This forum was perceived as beneficial, as many participants expressed how *‘comforting’* it was to talk to other families with autistic children, and also to read realistic experiences of others in similar situations.

*“I use the Facebook group a lot and I watch the chats and its helpful to see advice given for the future. It’s also nice to have that connection with other families who have an autistic child, it’s comforting.”*

*“Unlike a lot of social media, it’s not all just the positive highlight stuff, like people are putting on there, you know, not fails, but ‘this isn’t working’, ‘does anyone have any advice on this’, so it’s not a group that you go on where it’s just going to make you feel like you’ve been rubbish at training your dog, because actually, it’s a good balance.”*

## 4. Discussion

This qualitative study explored the experiences of families of children with ASC and their perceptions of the Family Dog Service and the perceived impact of human–dog interactions and relationships. Overall, findings indicate that companion dog ownership provided emotional and physical benefits and increased participation in activities and socialisation. Challenges associated with dog ownership were also discussed, but participants indicated that the guidance provided by the service helped to mitigate these difficulties and support the family. Parents appeared to have very positive perceptions of the service and were able to implement strategies and techniques they had learned from the workshops to harness the full potential of a companion dog for their child and wider family network.

### 4.1. Impact of Companion Dog on Families with ASC

The current findings indicate the potential of companion dogs to improve child wellbeing while also positively impacting the wider family network. While previous research has reported that an important factor of assistance dogs is the ability to provide more specific, specialist task work compared to untrained companion dogs (Gravrok et al., 2020); the benefits of a companion dog outlined in this study are similar to those reported for autism assistance dog placements (Burrows et al., 2008; Shoesmith et al., 2024). Based on the current findings and the previous literature evaluating the impact of companion dogs on families with ASC (Grandgeorge et al., 2012; Wright et al., 2015b), it is plausible to suggest that these benefits may not directly occur due to the specialist training that an assistance dog has received. Rather, these outcomes may be related to the presence of the dog and the relationship that develops between the dog, the child with ASC, and the wider family network. While the development of the relationship will occur naturally over time (Hellings et al., 2022), participants in the current study noted that the Family Dog Service provided the foundation to introduce the child and the dog in a way that would facilitate a healthy and positive relationship. It is possible that via the acquisition of a trained autism assistance dog, organisations provide specific support to both the individual and the dog, whereas breeders and shelters may not. This variation in support may potentially contribute to differences in the perceived impact of the dog in different contexts (Gravrok et al., 2020). This aligns with our current findings, as parents frequently valued the lifetime support and guidance offered via the Family Dog Service, starting from the consideration of acquiring a companion dog. While we do not suggest in any way that companion dogs can replace the important role played by trained assistance dogs, future research should explore the differences between families with assistance dogs, those with companion dogs alone, and those participating in services like the Family Dog Service. This could provide deeper insights into how dogs can impact families with ASC and the mechanisms underpinning these outcomes in various contexts.

Additionally, participants highlighted the key considerations when selecting a dog that may impact how the child with ASC interacts with and develops a relationship with the dog. For example, ensuring dog characteristics match the sensory preferences of the child (e.g., type and length of coat) or considering the size of the dog (e.g., larger dogs for deep pressure therapy). The current findings suggest that these are important considerations that may not have been at the forefront of their decision-making if participants had not received the support and guidance from the Family Dog Service. Future research should further explore how families with ASC make decisions when selecting the dog breed, and whether certain dog characteristics may impact the perceived benefits of dog ownership.

Lastly, despite these perceived benefits, participants in the current study also reported some challenges. These primarily included the time and commitment associated with dog ownership and training, aligning with previous evidence (Carlisle, 2014). While it is important for prospective owners to consider these factors before acquiring a dog, it appeared that the support and advice provided by the Family Dog Service mitigated any negative feeling towards these challenges and supported parents during the early stages of ownership and beyond. Overall, these findings highlight the importance of the service, which provides a unique and alternative approach to those who may be unable to access an autism assistance dog for a range of reasons.

### 4.2. Experiences and Perceptions of the Family Dog Service

The context in which dog owners may access or be exposed to opportunities for education and support regarding their dog varies considerably. This can be through formal methods or approaches (e.g., veterinary consultations or training programmes). However, it is more likely that owners access information via informal methods (e.g., television programmes, social media platforms). This information may be unregulated, inaccurate, and could potentially influence the way a dog is trained and subsequently the welfare of companion dogs living in family homes (Philpotts et al., 2019). Inaccurate information might be due to the lack of understanding or misinterpretation of information related to dog ownership, its complexities, and the existing evidence base. An example of this still remains clear in existing dog training programmes, with the concept of the ‘alpha dog’ and negative forms of punishment still being used and encouraged by self-appointed dog behaviour experts (Philpotts et al., 2019). In the current study, participants valued the Family Dog Service approach that discouraged these types of training methods and promoted the use of positive reinforcement, treating the dog as an equal member, and prioritising the dog’s welfare.

In addition to this positive approach, participants expressed how beneficial it was to find a service that was family-oriented and tailored to work specifically with ASC. This was commonly cited as one of the most appealing features of the service and also motivated most participants to initially engage with the workshops. Many parents expressed how difficult it was to find support to assist their child with ASC following a diagnosis, so the team’s expertise and knowledge in both dog training and living with ASC was highly valued. Given the heterogenous nature of ASC, it is imperative that any training delivered to parents of children with ASC adopts a person-centred approach, rather than enforcing a ‘one size fits all’ structure (Roberts et al., 2023). This tailored approach was also reflected in the ongoing lifetime support offered by the Family Dog Service team, who acknowledge ASC is not a static condition, and every child will have unique experiences and challenges. This understanding of how ASC impacts the child and their family appeared to be a key factor for successful uptake and implementation.

The interactive nature of the workshops and access to a private Facebook group that offered the opportunity to communicate with other parents in a similar situation was also well received. Support from other parents was perceived as beneficial for both aspects related to dog ownership and difficulties associated with their child’s confirmed or suspected diagnosis. This aligns with previous research, as studies have reported that parents who received support from their peers felt heard, validated, and connected to others who have shared similar experiences (Batchelor et al., 2021; Lee et al., 2024; Sullivan-Bolyai & Lee, 2011). The support offered by other parents, alongside the ongoing support offered by the Family Dog Service team, also appeared to be a key facilitator for successful engagement.

### 4.3. Limitations

Firstly, self-selection bias may influence the current findings as parents who agreed to participate may have had different experiences than those who chose not to participate. It may be the case that those who had a positive experience of the service and/or introducing a companion dog to the home were more willing and motivated to express interest in participating. Therefore, the findings may not be generalisable to the entire cohort who have experienced the Family Dog Service. In the future, a larger sample would be required to demonstrate the effectiveness of the service, and whether the service results in positive impacts that may not be seen as a result of a companion dog without the additional support and guidance. Secondly, the sample was primarily limited to the inclusion of mothers as only one father opted to participate. Fathers of children with ASC have been underrepresented in the literature and their perspectives may differ (Adkins et al., 2023). This gender imbalance is a commonly cited barrier in human–animal interaction research, and future, larger studies should actively seek to overcome this, e.g., by stratified sampling strategies. Thirdly, the service is accessible to families with children with ASC across a broad age range (3–16 years). While this is important for ensuring the service remains accessible to a diverse demographic of families with children with ASC, this range may also introduce variations in the benefits and challenges experienced by parents of young children compared to those of adolescents. Although no such differences were observed in the current data, further research with a larger sample of participants could provide valuable insights into whether distinct differences occur across different age groups. Lastly, interviews were only conducted with parents who had engaged with the Family Dog Service online since it was adapted from in-person workshops due to the COVID-19 pandemic. It would be beneficial to explore the benefits and challenges of this method of delivery and compare experiences to those who engaged with the service in person.

## 5. Conclusions

Companion dogs can have a positive impact on children with ASC and may be an important source of support for the wider family network. However, to harness the full potential of a companion dog, it is important for families to have access to educational and support programmes that provide guidance and strategies to facilitate a healthy, positive, and supportive relationship between the dog and the family. While the findings do not claim that companion dogs can replace the important role played by specialist trained assistance dogs, this service may provide a practical and valuable alternative in light of the restrictions and challenges often associated with securing assistance dog placements.

## Figures and Tables

**Table 1:** Overview of Family Dog Service live workshops.

Workshop	Content
Introduction	The introduction involves meeting other people in the group, the instructors, and is for the Dogs for Good team to ensure the group is comfortable and familiar with the technical aspects of the workshops.l
Selecting the right dog	This workshop is optional and covers considerations regarding the type of dog to select (e.g., breed, size, age) and advice on buying or adopting a dog (e.g., from rescue centres or breeders).
Workshop 1	This workshop involves information related to looking after the family dog (including meeting their needs). This includes guidance on food, nutrition and diet, health (including how to perform a basic health check), and considerations around grooming. This workshop also involves content related to safety in the home and appropriate behaviour, positive mental experiences for their dog in their experience of growing and learning, dog-related laws, building the dog and child relationship, and considering ways to support both the child and the dog.
Workshop 2	This workshop primarily focuses on how families can help their dog in the home to make sure they feel safe and comfortable. Content includes understanding dog language and communication, what to do with the dog’s communication, how the dog can help the child, the theory of how dogs learn, how to encourage wanted behaviour, and the impact of negative methods on the relationship with the family dog.
Workshop 3	This workshop involves content relating to how to train the dog, discussing different dog training styles and tools, and advanced training and recognised autism assistance taskwork (e.g., head rest, body rest, nose nudge, button push).

**Table 2:** Interview participant characteristics.

		N (%)
Gender	Female	15 (93.8)
	Male	1 (6.2)
	35–44	9 (56.3)
	45–54	7 (43.7)
Ethnicity	White (any White background)	15 (93.8)
	Chinese	1 (6.2)
Number of children with ASC ^1^	1	10 (62.5)
	2	5 (31.3)
	5	1 (6.2)
Age of children with ASC ^1^	3–6	3 (12.0)
	7–10	11 (44.0)
	11–14	9 (36.0)
	15–16	2 (8.0)
Gender of children with ASC ^1^	Female	9 (36.0)
	Male	16 (64.0)
Comorbidities of children with ASC ^1^	Anxiety disorder	6 (37.5)
	ADHD	6 (37.5)
	Learning difficulties (e.g., dyslexia, dyspraxia)	4 (25.0)
Family dog breed	Labrador	4 (25.0)
	Labradoodle	2 (12.5)
	Golden doodle	2 (12.5)
	Whippet	1 (6.3)
	Dachshund	1 (6.3)
	Staffordshire Bull Terrier	1 (6.3)
	Cocker Spaniel/Poodle mix	1 (6.3)
	Springer Spaniel/Poodle mix	1 (6.3)
	Cocker Spaniel/Yorkshire Terrier mix	1 (6.3)
	Beagle	1 (6.3)
	St. Bernard	1 (6.3)
Length of current dog ownership	6 months–1 year	2 (12.5)
	1–3 years	8 (50.0)
	3 years+	6 (37.5)
Previous experience of dog ownership	Yes	10 (62.5)
	No	6 (37.5)

^1^ Confirmed or suspected diagnosis.

**Table 3:** Themes and associated sub-themes.

**Themes**	**Sub-Themes**
Improvement of psychosocial and functional aspects associated with ASC	Enhanced focus and attention Reduction in loneliness and engagement in social interaction Increased physical exercise and time outdoors Reduced anxiety-based behaviours Tolerating changes and willingness to engage in new activities
Positive impact of the companion dog on parents of children with ASC	Increased parental respite Increased parental wellbeing
Challenges and considerations associated with dog ownership for families with children with ASC	Key considerations when selecting a dog Adjusting to a new dog within the family unit Challenges associated with the development of the human–dog relationship
Experiences of the Family Dog Service workshops	Motivation to attend the workshops Information tailored to families with ASC Access to lifetime support

## Data Availability

All relevant data are uploaded to the OSF repository and available via the following URL: https://osf.io/6m3rh/ (accessed on 2 June 2024).

## Supplementary Materials

The following supporting information can be downloaded at: https://www.mdpi.com/article/10.3390/bs15020162/s1, S1: Interview topic guide.
