# Supplementary material for: Supporting Families to ‘Make the Most’ of the Relationship Between Their Companion Dogs and Their Children with Autism Spectrum Condition: A Qualitative Exploration of the ‘Family Dog Service’
Source: Behav Sci (Basel). 2025 Feb 1;15(2):162. doi: 10.3390/bs15020162 (PMC11851659; doi:10.3390/bs15020162)
Supplement: Supplementary file 1 [file behavsci-15-00162-s001.zip › behavsci-3385367-supplementary.pdf]

Supplementary Material S1. Interview topic guide

1. **Could you tell me a little bit about yourself and your family?**
    a. Can you tell me a little bit more about your child(ren) with ASC?
    b. Can you tell me a little more about how this has influenced or impacted your day to day activities?

2. **What made you decide to buy/adopt a dog?**
    a. Is this the first family dog you have owned?
    b. What breed is your dog? What influenced your decision to select this specific breed?
    c. Who did you seek advice from (if anyone) when deciding to buy/adopt a dog?
    d. How did you go about buying/adopting your dog?
    e. What are the challenges you encountered when choosing to adopt/by a dog?
    f. What were your goals/expectations for you and your child(ren) when deciding to buy/adopt a dog?

3. **How did you hear about the Family Dog Service?**

4. **How have you engaged with the Family Dog Service?**
    a. What influenced your decision to attend any workshops?
    b. Which workshops have you attended (and when)?
    c. What part of the workshops were most important/helpful to you? What is it about the DfG training that seems most helpful (maybe compared to how they would have otherwise trained a new dog)?
    d. What were your least favourite parts of the workshops/least helpful to you?
    e. Is there any advice/training you wish you could have had to: *(where applicable)* improve decision making when adopting/buying a dog, integrating your family dog into your home/assisting with living with your family dog?
    f. Overall, how has the service helped you and your family? Would they recommend the training, and in a nutshell, why?

5. **How have you found working with the Dogs for Good team?**
    a. How have you found the contact with the Dogs for Good team when seeking advice?
    b. Has the ongoing advice and support been beneficial for you and your family? How?
    c. What are the main reasons you have decided to seek advice/support from the team?

6. **Have you used the Family Dog Facebook group to seek advice?**
    a. Do you primarily use the group to seek advice from others in similar situations/DfG team (or both)?
    b. Have you met others via the Facebook group that you have built a relationship with?
    c. How has the Facebook group helped you?
    d. What type of advice is (or was) particularly helpful for you? *Prompt: advice around consideration of adopting/buying a dog, integrating the dog into family unit, training for specific ASC needs, practical advice.*

7. **Please could you tell me a little bit more about your family dog and the challenges/benefits you have encountered when integrating the family dog into your home?**
    a. What are the challenges/benefits when integrating your family dog into your family life? *Prompts: difficulties/benefits of temperament and behaviour; development of bond; difficulties/benefits of dogs skills to work with child*
    b. Please describe your experience of adapting your lifestyle/routine and the 'settling in' period.
    c. What was your child's experience of integrating your dog into the family home?

   d. Do you feel your child(ren) with ASC bonded with your family dog when they first arrived?
   e. Do you feel the family has bonded with your family dog when they first arrived?
   f. *Where applicable*, if a new dog due to loss of previous dog, could you please describe how you/your child(ren) have dealt with feelings of grief?

8. What are the challenges/benefits you have encountered since your family dog has settled into your home?
   a. Have you had any continued challenges from when you initially brought the family dog home? Are there any new challenges?
   b. What are the main benefits of your family dog?

9. (If applicable) Thinking about these challenges, has the Family Dog Service mitigated any of these?
   a. If so, how? If not, why not?
   b. Do you think integrating your family dog into your home would have been different without the Family Dog Service? If so, how/why?

10. How has your family dog impacted your child/ren with ASC?
   a. How/in what way has the dog changed behaviour or dynamics in the family? What exactly do you think it is about the dog that has provided any change? Bond, relationship, other? Has the training helped this?
   b. Have you noticed any changes in:
      i. Social and emotional skills
      ii. Sense of independence/sense of self
      iii. Calmness and effect on anxiety-based behaviour
      iv. Physical activity (exercise/motor skills)
      v. Attention, concentration and focus (activities with/without dog)
      vi. Language skills (verbal and non-verbal)
      vii. Interaction with local community
      viii. Any unexpected benefits?
   c. Has the dog met your expectations/goals when you initially decided to integrate a family dog into your home?

11. Could you tell me a little bit more about your child(ren)'s bond/relationship with the family dog?
   a. How would you describe your child(ren)'s bond with the family dog now? How important has this bond been to your child?
   b. What is the relationship between their child and the dog? Can they describe it? What makes it special, and how did the training help with any of this?

12. Could you explain a little more about how your dog has assisted your child(ren)?
   a. Did you train your family dog to specifically assist in these areas?
   b. Are there any unexpected things your dog does that assists your child?

13. What impact has your dog had on you and/or your other relatives?
   a. How has your dog impacted you/other relatives (e.g., spouse/siblings?)
   b. Have there been any unexpected benefits directly for you or on your overall family life?
   c. How would you describe your bond with the family dog? How important has this been to you?

14. Is there anything else you would like to discuss about your family dog or the Family Dog Service that we haven’t covered today?
